# Mental Health Disorders and Summer Temperature-Related Mortality: A Case Crossover Study

**DOI:** 10.3390/ijerph17239122

**Published:** 2020-12-07

**Authors:** Elisa Stivanello, Federico Chierzi, Paolo Marzaroli, Sara Zanella, Rossella Miglio, Patrizia Biavati, Vincenza Perlangeli, Domenico Berardi, Angelo Fioritti, Paolo Pandolfi

**Affiliations:** 1 Department of Pubblic Health, Azienda USL di Bologna, 40121 Bologna, Italy; paolo.marzaroli@ausl.bologna.it (P.M.); patrizia.biavati@ausl.bologna.it (P.B.); vincenza.perlangeli@ausl.bologna.it (V.P.); paolo.pandolfi@ausl.bologna.it (P.P.); 2 Department of Mental Health, Azienda USL di Bologna, 40123 Bologna, Italy; federico.chierzi@ausl.bologna.it (F.C.); angelo.fioritti@ausl.bologna.it (A.F.); 3 Department of Statistical Sciences, University of Bologna, 40126 Bologna, Italy; sara.zanella3@studio.unibo.it (S.Z.); rossella.miglio@unibo.it (R.M.); 4 Department of Medical and Surgical Sciences (DIMEC), University of Bologna, 40123 Bologna, Italy; domenico.berardi@unibo.it

**Keywords:** high temperature, mental health, vulnerability, heat waves

## Abstract

Identifying the most vulnerable subjects is crucial for the effectiveness of health interventions aimed at limiting the adverse consequences of high temperatures. We conducted a case crossover study aimed at assessing whether suffering from mental health disorders modifies the effect of high temperatures on mortality. We included all deaths occurred in the area of Bologna Local Health Trust during the summers 2004–2017. Subjects with mental disorders were identified by using the local Mental Health Registry. A conditional logistic model was applied, and a z-test was used to study the effect modification. Several models were estimated stratifying by subjects’ characteristics. For every 1 °C above 24 °C, mortality among people without mental disorders increased by 1.9% (95% CI 1.0–2.6, *p* < 0.0001), while among mental health service users, mortality increased by 5.5% (95% CI 2.4–8.6, *p* < 0.0001) (z-test equal to *p* = 0.0259). The effect modification varied according to gender, residency and cause of death. The highest probability of dying due to an increase in temperature was registered in patients with depression and cognitive decline. In order to reduce the effects of high temperatures on mortality, health intervention strategies should include mental health patients among the most vulnerable subjects taking account of their demographic and clinical characteristics.

## 1. Introduction

High summer temperatures are now a known threat to human health. Extensive research has shown that summer heat waves and high temperatures are associated with an excess mortality attributable to several causes [1,2,3]. The effects of high temperatures on human health and wellbeing are a major public health concern, especially in the context of climate change. As the adverse health effects of heat are partially preventable [4], public health interventions will be of paramount importance for responding to the climate and population changes expected to take place in the coming years [5].

Vulnerability to high summer temperatures varies according to geographical, environmental and individual factors [1,6,7]. However, while the relationship between excess heat and mortality is well known, the research published on heat-vulnerable subgroups is not fully conclusive [2]. Nonetheless, increasing awareness of the most vulnerable subgroups is crucial for the success of public health programs [8] and is necessary for the implementation of specific intervention strategies [2].

Previous studies found that people with lower socio-economic status, living alone, the elderly and those with pre-existing chronic diseases such as cardiovascular and respiratory diseases or diabetes [2,3,7,9,10] are more vulnerable to heat and have higher temperature-associated mortality risk than people without these conditions. Mental disorders were suggested to be correlated with heat vulnerability [11]. However, only a few studies have investigated the impact of extreme heat on mortality in a sample of patients with mental illnesses [12,13]. In particular, there is still a lack of knowledge regarding the impact of high temperatures on patients affected by depression or other common mental disorders [14]. Almost one billion people currently suffer from mental disorders worldwide, placing mental illnesses among the leading causes of the Global Burden of Disease [15]. Depressive disorders are one of the five leading causes of Years Lived with Disability (YLD). It is well known that there is a mortality gap between the general population and people suffering from mental illnesses [16,17]. 

We thus conducted a retrospective population-based study with a case-crossover analysis, aiming, firstly, to determine whether suffering from mental disorders impacts on the risk of death during hot days and, secondly, to explore temperature-related mortality risk according to patient characteristics, causes of death and psychiatric diagnoses.

## 2. Materials and Methods 

### 2.1. Setting and Population

The study was carried out at the Local Health Trust of Bologna (LHT), which covers a catchment area of 2915 km^2^ with more than 880,000 inhabitants in northern Italy. Within the LHT, the Bologna Mental Health Department (MHD) is responsible for managing and planning all medical and social activities related to the prevention, treatment and rehabilitation of mental health disorders across the area as previously described [17]. 

We included all subjects aged 18 or older, resident in Bologna LHT catchment area who died during the summer period (15th May–30th September) of the years 2004–2017.

The study population was identified by using the Bologna Registry of the Causes of Death which covers all the deaths in the catchment area and provides information about socio-demographic characteristics, including residency, date of birth and the date, circumstances, place and causes of death according to the ICD9 (International Classification of Diseases) (until 2009) or the ICD10 (after 2009) (Table S1 in Supplementary Materials for ICD9 and ICD10 codes corresponding to each cause of death). In order to identify people with mental disorders, we linked the Bologna Registry of the Causes of Death with the MHD case registry relative to the 2004–2017 period. The MHD case registry covers all psychiatric outpatient consultations and activities of the MHD. It contains information on date of consultations, treatment and ICD-9CM diagnoses. The information is entered at the point of care and then collected at the LHT level. 

We linked the two registries deterministically by using the tax code, an identification code that is unique for every resident of Italy and that is reported in administrative and health documents. Afterwards, the database was anonymized and the population was divided into two groups:

1. All subjects who had accessed the services of the MHD at least once (the MHD population);
2. All subjects who had never accessed the services of the MHD (the non-MHD population).

We excluded patients who had accessed the services of the MHD but had not been given any psychiatric diagnosis (codes ICD-9-CM ≥ 290 e ≤ 319). 

The study was approved by the Area Vasta Emilia Centro Ethical Committee (n. 339, 2019). The STROBE (Strengthening the Reporting of Observational Studies in Epidemiology) guidelines were followed for this manuscript. 

### 2.2. Outcomes and Environmental Variables

The main outcome of the study was deaths for all causes; secondary outcomes were deaths due to natural, external causes and causes categorized according to the ICD chapters (Table S1 in Supplementary Materials).

The exposure variable was the mean apparent daily temperature (Tapp). This was calculated as Tapp = −2.653 + (0.994 × mean temperature) + (0.0153 × T_d_^2^), where T_d_ is dew point, calculated as Td = (237.7 × a)/(17.27 − a), where a = ((17.27 × T)/237.7 + T)) + lb(mean humidity/100).

Data on temperature (T), dew point and humidity were collected in 18 monitoring stations of the Regional Agency for Prevention, Environment and Energy of Emilia Romagna. Subjects living in municipalities (the basic administrative entity in Italy) with a monitoring station were attributed the data of this station, whereas subjects living in municipalities without stations were attributed data collected in the nearest monitoring station located at a similar altitude (Table S2 in Supplementary Materials). 

We also collected daily data on ozone (daily maximum 8-h running mean) from 3 stations of the Regional Agency for Prevention, Environment and Energy of Emilia Romagna.

### 2.3. Statistical Analysis

To study the association between temperature (same and previous day) and mortality, we applied the case-crossover approach, a method proposed to study triggers of acute events and largely used in air pollution epidemiology [18]. In this approach, each individual serves as its own control and therefore encompasses the need to adjust for time-invariant confounders. Control periods were selected using the time-stratified method in which each case was matched to controls on the same day of the week in the same month and year. Firstly, we explored the concentration response curve of the relationship between apparent temperature and mortality. A conditional logistic regression analysis was performed, modelling the exposure variable as a cubic penalized spline. The level of apparent temperature when mortality assumes a steep linear trend was inspected [19] in order to find the optimal cut-off point to interpolate this relationship with two line segments. Secondly, we estimated the probability of dying in the two populations (the MHD population and the non-MHD population) by applying a conditional logistic model adding the time-variant variables, namely, the ozone at lag 0–1 and the temporary population decrease in the summer period (2 weeks in August). We added two linear terms in the model for the exposure variables. The first term refers to the mean apparent temperature at lag 0–1 and shows the change in risk of mortality for a 1 °C increase. The second term refers to the additional effect of a 1 °C increase above the cut-off. The final effect of the temperature on mortality over the cut-off, reported as an OR (odds ratio), is calculated by summing the effect of both terms.

To study the effect modification of belonging to the MHD population on the probability of dying and to test the hypothesis of equal effect of temperature in the two populations, a z-test was used [20]. The model was repeated with the secondary outcomes (natural and external causes of death) and replicated stratifying by belonging or not to the MHD population, and by gender, age-class, residency (urban vs rural) and psychiatric diagnosis grouped according to Lora et al. [21] (Table S3 in Supplementary Materials). 

Statistical analyses were conducted using STATA 12.1, StataCorp, College Station, Texas, TX, USA.

## 3. Results 

The mean apparent temperature during the study period was 21.48 °C (±4.57) and the mean temperature 20.85 °C (±4.03). Table 1 presents a summary of the environmental variables considered in the study.

A total of 48,305 deaths occurred during the study period: 45,278 had never had any access to the MHD, and 3027 had had at least one access to the MHD, of which 19 were excluded because they did not have a psychiatric diagnosis. 

**Figure 1:**
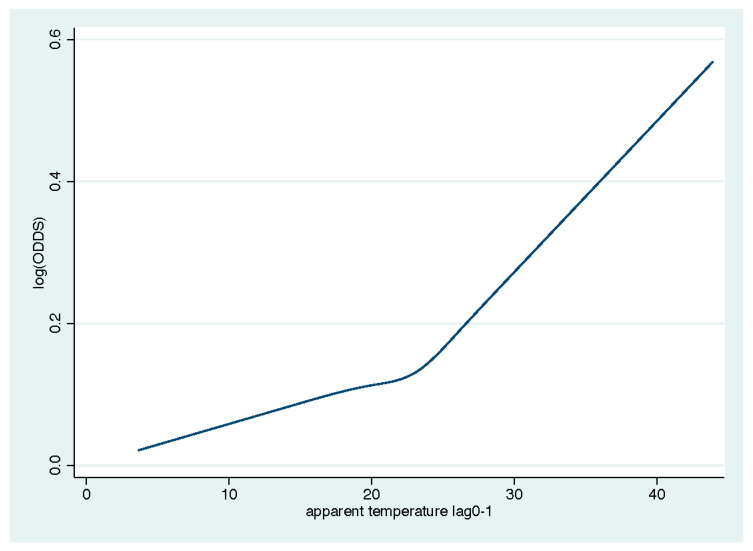
Prediction of death in the study populations according to apparent temperature at lag 0–1.

Figure 1 shows the concentration–response curve between daily mean apparent temperature (lag 0–1) and mortality. The curve is J-shaped with a turning point at 24 °C, this cut-off value reports the highest log likelihood among a set of potential values. 

The MHD population was younger, with a higher percentage of women and residents in rural areas than the non-MHD population (see Table 2). 

For every 1 °C above 24 °C, in the non-MHD population, mortality increased by 1.9% (95% CI 1.2–2.6%, *p* < 0.001) whereas in the MHD population it increased by 5.5% (95 CI% 2.4–8.6, *p* < 0.001). Belonging to the MHD population modifies the effect of temperature on mortality (*p* value of the z-test equal to 0.026). In the non-MHD population only natural mortality increases with temperature, in the MHD population both natural and external causes of death increase with temperature (Table 3). 

In the non-MHD population temperature is significantly associated with an increase in the odds of dying due to neoplasms, diseases of the nervous, circulatory and respiratory system. Being in the MHD population increased the odds of mortality for neoplasms associated with temperature (*p* value of the z-test equal to 0.043). 

Table 3 also shows ORs of dying according to demographic characteristics. Males and residents in rural area belonging to the MHD population have higher odds of dying per 1 °C above 24 °C than males and residents in rural areas belonging to the non-MHD population (*p* < 0.05 in the z-test). Within the MHD population, males and residents in rural areas also have higher odds of dying per 1 °C above 24 °C than females or residents in urban areas although the *p* value in the z-test is >0.05 (0.123, 0.296, respectively; data non shown in tables).

Within the MHD population, the highest probability of dying due to an increase in 1 °C above 24 °C was registered in patients with a diagnosis of depression (OR: 1.083, 95% CI 1.03–1.138, *p* = 0.002) and of dementia and cognitive decline (OR: 1.074, 95% CI 1.022–1.129, *p* = 0.005). The OR was greater than 1 in patients with psychosis or mania and bipolar affective disorders but did not reach significance (Table 4).

## 4. Discussion

This study confirms that high summer temperatures increase the risk of death but shows also that the Mental Health Services Users of Bologna are more vulnerable to high temperatures than the general population, a finding that is in line with the limited existing literature on this subject [11,13,20]. Moreover, we found different probabilities of dying associated with summer temperatures depending on demographic and clinical characteristics. Several factors might explain why people affected by mental disorders could be particularly vulnerable to adverse ambient conditions like high temperatures [11]. Chronic health conditions impairing thermoregulatory responses to heat stress, like cardiovascular, respiratory diseases and diabetes [22] are highly prevalent among mental health patients [23]. In addition, people suffering from mental illnesses might have limited coping mechanisms when faced with high temperatures, such as finding cold environments, wearing appropriate clothing and drinking more water [22]. Some studies [24] have demonstrated that people with mental disorders have problems with access to healthcare which could have a particularly negative impact during heat waves. Psychotropics could also play a role [25,26], as suggested by a recent study [13] reporting that the use of antipsychotics and hypnotics is strongly related to the risk of death during hot weather in people with psychosis, dementia and substance misuse.

Interestingly, we found that being a MHD patient modifies vulnerability to temperature within some categories. MHD patients that are male or residents in rural areas have significantly higher temperature-related mortality odds than the corresponding groups in the non-MHD population, probably because they suffer the effects of numerous vulnerable factors. 

In regards to place of residence, the rural areas of Bologna LHT are often characterized by higher deprivation, limited access to health services and a less healthy lifestyle culture [27,28] than urban areas. These contextual factors do not affect the health of all equally [29] and could be particularly detrimental to patients suffering from mental illnesses exacerbating individual disadvantages and elevating their risk of death as a consequence of high temperature. 

With regard to causes of death, mortality due to external causes increases with temperature in the MHD. This was not the case with the non-MHD population. Subjects suffering from mental disorders could be especially prone to heat-induced impulsive and disinhibited behaviors or to a reduction in cognitive skills leading to an increase of fatal accidents and other external causes [30,31]. This group of causes includes intentional self-harm which is a well-known outcome associated with increases in temperatures [32]. 

As widely reported in previous studies [1,3], we confirmed the association between elevated temperatures and cardiovascular and respiratory mortality. This association was stronger for the non-MHD population than for the MHD population, who showed a significantly higher risk of dying from neoplasms than the rest of the population. This could be explained through considering that over the last two decades increasing attention has been paid to cardiovascular illnesses in patients with mental diseases [33]. Whereas different pre-existing diseases such as neoplasms were less likely to be addressed by public health policies. In addition, there is evidence that people with mental illnesses still face difficulties in attending cancer screenings [34,35] and in accessing specialized treatments [36,37] and, therefore, may present a severe clinical picture at diagnosis that might be exacerbated during adverse ambient conditions such as elevated temperatures. 

Last but not least, our data shows that the association between temperature and mortality is not significant in all psychiatric diagnoses, and this could be partly due to the small number of patients affected by some diagnoses. Nevertheless, it is worth noting that MHD users with depression carry the highest risk of temperature-related mortality. Depressed patients are generally expected to have higher social functioning than patients with schizophrenia or other psychoses and so are not considered to be part of the at risk population; therefore, they could be excluded from heat prevention interventions [4]. However, since there is evidence that people with depression may have a primary dysfunction of thermosensory/thermoregulatory cooling mechanisms [38,39], they may experience elevated body temperature in response to heat [38]. Moreover, dopamine/noradrenaline reuptake inhibitors have been shown to significantly increase core temperature in humans during physical efforts [26], and selective serotonin reuptake inhibitors (SSRIs) seem to disturb thirst perception, enhancing the risk of drug-induced hyponatremia during the hot season [26]. A recent study found that antidepressants were not associated with an enhanced risk of dying during hot weather [13]; nonetheless, further work is needed in order to understand how antidepressants may impair physiological responses to extreme heat [13].

This study provides evidence on mental health patient vulnerability to high temperatures. One of the strengths of this study is that we were able to explore vulnerability to heat in both severe and non-severe mental diseases since we had access to a mental health registry that covers all subjects treated by the public mental health services. 

Another strength of the study is related to its design. By using case crossover design, individual confounders are controlled and strong residual confounding due to unmeasured time-dependent covariates is unlikely. In addition, the exposure variables were included in relation to two terms, one referring to the change in risk of mortality at each 1 °C increase and one referring to the change in risk of mortality due to the number of degrees above the cut-off.

One limit of the study is the possible measurement errors due to the fact that personal exposure may differ from the environmental values estimated by monitoring stations. Indeed, there might be differences in topography and altitude between the place of residence of the subjects and the fixed monitors. In a sensitivity analysis, excluding all subjects residents in the municipalities of the mountain area where the differences in altitude between residence and station are likely greater, we had similar results (data not shown). Nevertheless, our assessment of exposure was more likely subject to nondifferential misclassification that is expected to attenuate the effect estimates. 

Another limit is the difference in size of the two populations and the small size of some categories. The analyses by strata do not always have enough statistical power and should be considered with caution and further investigated in future research. Further research is also needed to understand why some subgroups of patients (e.g., patients with neoplasms, males and patients living in rural areas) are more disadvantaged and also to understand the role played by substance abuse.

## 5. Conclusions

Our study confirms the impact of temperature on mortality and shows that patients with mental disorders are more exposed to the risk of dying due to extremely high temperature than the rest of the population highlighting that this risk could vary according to individual characteristics like psychiatric diagnosis, sex and place of residence. 

In recent years, several public programs have been promoted to prevent the health consequences of heat. In order to improve their effectiveness, public health prevention plans should recognize people affected by mental disorders as being among the most vulnerable subjects during heat waves or high summer temperature days, and attention should be given not only to severe and chronic disorders but also to common mental health disorders such as depression.

## Figures and Tables

**Table 1:** Environmental variable statistics.

	Apparent Temperature (°C)	Mean Temperature (°C)	Humidity	Ozone, mcg/m^3^
mean	21.48	20.85	61.9	108.75
sd	4.57	4.03	11.47	26.02
minimum	6.75	4.63	18	10.5
maximum	31.9	34.02	100	229.62
percentiles				
25th	18.27	17.75	52	84.75
50th	21.65	21.02	62	104.88
75th	25.09	24.19	71	126.38
% missing	9.15	0.66	9.15	8.86

sd: standard deviation.

**Table 2:** Characteristics of the study populations by demographic characteristics, specific cause of death and psychiatric diagnosis.

	Non-MHD		MHD	
	*n*	%	*n*	%
**Total**	45,278	100	3008	100
**age class**				
18–40	537	1.19	76	2.53
41–64	4005	8.85	557	18.52
65–74	6066	13.40	527	17.52
75–84	13,805	30.49	967	32.15
>84	20,865	46.08	881	29.29
**gender**				
male	21,266	46.97	1312	43.62
female	24,012	53.03	1696	56.38
**cause of death**				
Natural	43,251	95.52	2741	91.12
Certain infectious and parasitic diseases	908	2.01	94	3.14
Neoplasms	14,712	32.61	693	23.14
Diseases of the blood and blood-forming organs and certain disorders involving the immune mechanism	231	0.51	10	0.33
Endocrine, nutritional and metabolic diseases	1772	3.93	155	5.18
Mental and behavioral disorders	1781	3.95	212	7.08
Diseases of the nervous system	1404	3.11	153	5.11
Diseases of the circulatory system	15,610	34.60	910	30.38
Diseases of the respiratory system	3271	7.25	261	8.71
Diseases of the digestive system	1650	3.66	112	3.74
Diseases of the skin and subcutaneous tissue	69	0.15	9	0.30
Diseases of the musculoskeletal system and connective tissue	200	0.44	16	0.53
Diseases of the genitourinary system	831	1.84	63	2.10
Pregnancy, childbirth and the puerperium	4	0.01		
Certain conditions originating in the perinatal period	2	0.00		
Congenital malformations, deformations and chromosomal abnormalities	43	0.10	7	0.23
Symptoms, signs and abnormal clinical and laboratory findings, not elsewhere classified	605	1.34	33	1.10
External causes of morbidity and mortality	2028	4.49	267	8.91
**residency**				
urban	26,562	58.66	1552	51.60
rural	18,716	41.34	1456	48.40
**psychiatric diagnosis**				
Schizophrenia and other functional psychosis			335	11.14
Mania and bipolar affective disorders			147	4.89
Depression			1039	34.54
Neurotic disorders			309	10.27
Disorders of personality and behavior			156	5.19
Alcoholism and substance abuse			49	1.63
Dementia and cognitive decline			939	31.22
other			34	1.13

Non-MHD: All subjects who had not accessed the MHD (Mental Health Department). MHD: All patients who had accessed the MHD at least once.

**Table 3:** OR (odds ratio) of dying per 1 °C increase above 24 °C in Mean Apparent Temperature (lag 0–1) and 95% confidence interval (CI) in the study populations by age, gender, cause of death, residency and *p* value of the z-test.

	Non-MHD				MHD				z-Test
	OR	95% CI		*p*	OR	95% CI		*p*	*p*
**Total**	1.019	1.012	1.026	<0.001	1.055	1.024	1.086	<0.001	0.0255
**age class**									
18–40	1.039	0.969	1.113	0.283	1.063	0.865	1.307	0.562	0.834
41–64	0.99	0.963	1.018	0.476	1.072	0.997	1.152	0.059	0.044
65–74	1.007	0.985	1.029	0.531	1.065	0.992	1.142	0.081	0.137
75–84	1.024	1.009	1.039	0.001	1.048	0.995	1.104	0.074	0.393
>84	1.023	1.011	1.035	<0.001	1.046	0.992	1.103	0.094	0.415
**gender**									
male	1.017	1.006	1.029	0.004	1.083	1.036	1.132	<0001	0.007
female	1.020	1.009	1.031	0.000	1.034	0.993	1.075	0.102	0.518
**cause of death**									
Natural	1.020	1.011	1.028	0.000	1.052	1.020	1.085	0.001	0.053
Certain infectious and parasitic diseases	1.020	0.973	1.068	0.410	0.974	0.839	1.130	0.727	0.563
Neoplasms	1.013	1.001	1.025	0.039	1.072	1.016	1.131	0.011	0.043
Diseases of the blood and blood-forming organs and certain disorders involving the immune mechanism	1.055	0.988	1.203	0.086	1.176	0.770	1.797	0.452	0.731
Endocrine, nutritional and metabolic diseases	1.015	0.967	1.036	0.973	1.113	0.991	1.250	0.072	0.086
Mental and behavioral disorders	1.018	0.984	1.053	0.305	1.090	0.996	1.194	0.061	0.162
Diseases of the nervous system	1.062	1.024	1.102	0.001	1.088	0.973	1.217	0.138	0.683
Diseases of the circulatory system	1.032	1.020	1.044	<0.001	1.039	0.994	1.087	0.093	0.759
Diseases of the respiratory system	1.034	1.008	1.060	0.010	1.015	0.929	1.110	0.740	0.705
Diseases of the digestive system	0.999	0.963	1.036	0.966	1.011	0.891	1.147	0.864	0.806
Diseases of the skin and subcutaneous tissue	1.204	0.992	1.460	0.060	0.863	0.466	1.597	0.639	0.312
Diseases of the musculoskeletal system and connective tissue	1.024	0.925	1.133	0.653	1.262	0.891	1.787	0.190	0.739
Diseases of the genitourinary system	1.010	0.963	1.060	0.678	0.979	0.817	1.173	0.816	0.258
Pregnancy, childbirth and the puerperium	1.167	0.622	2.191	0.631					
Certain conditions originating in the perinatal period	0.000			0.998					
Congenital malformations, deformations and chromosomal abnormalities	0.977	0.805	1.185	0.814	0.000			0.997	0.997
Symptoms, signs and abnormal clinical and laboratory findings, not elsewhere classified	0.994	0.939	1.052	0.842	1.059	0.811	1.383	0.674	0.844
External causes of morbidity and mortality	0.993	0.962	1.025	0.671	1.096	1.007	1.194	0.034	0.273
**residency**									
urban	1.023	1.013	1.034	<0.001	1.049	1.004	1.095	0.031	0.283
rural	1.012	1	1.024	0.057	1.057	1.015	1.101	0.008	0.044

Non-MHD: All subjects who had not accessed the MHD (Mental Health Department); MHD: All patients who had accessed the MHD at least once.

**Table 4:** Odds Ratio (OR) of dying per 1 °C increase above 24 °C in Mean Apparent Temperature (lag 0–1) and 95% confidence interval (CI) by psychiatric diagnosis.

Psychiatric Diagnosis	OR	95% CI	*p*
Schizophrenia and other functional psychosis	1.048	0.951–1.156	0.343
Mania and bipolar affective disorders	1.027	0.881–1.197	0.731
Depression	1.083	1.030–1.138	0.002
Neurotic disorders	0.985	0.897–1.083	0.761
Disorders of personality and behavior	0.957	0.835–1.098	0.532
Alcoholism and substance abuse	0.960	0.715–1.290	0.788
Dementia and cognitive decline	1.074	1.022–1.129	0.005
Other	0.902	0.672–1.211	0.492

## Supplementary Materials

The following are available online at https://www.mdpi.com/1660-4601/17/23/9122/s1, Table S1: ICD 9 and ICD 10 for the classification of the causes of death, Table S2: Classification, altitude, area and population by municipality and monitoring station attributed to each municipality with its altitude and location, Table S3: Psychiatric diagnosis group according to ICD9-CM.
