# Supplementary material for: Mental Health Disorders and Summer Temperature-Related Mortality: A Case Crossover Study
Source: Int J Environ Res Public Health. 2020 Dec 7;17(23):9122. doi: 10.3390/ijerph17239122 (PMC7731125; doi:10.3390/ijerph17239122)
Supplement: Supplementary file 1 [file ijerph-17-09122-s001.pdf]

Supplementary Materials

Table S1. ICD 9 and ICD 10 for the classification of the causes of death.

Causes of death	ICD9	ICD10
Natural	001-799	A00-R99
Certain infectious and parasitic diseases	001-139	A00-B99
Neoplasms	140-239	C00-D48
Diseases of the blood and blood-forming organs and certain disorders involving the immune mechanism	240-279	D50-D89
Endocrine, nutritional, and metabolic diseases	280-289	E00-E90
Mental and behavioral disorders	290-319	F00-F99
Diseases of the nervous system*	320-389	G00-H99
Diseases of the circulatory system	390-459	I00-I99
Diseases of the respiratory system	460-519	J00-J99
Diseases of the digestive system	520-579	K00-K93
Diseases of the skin and subcutaneous tissue	680-709	L00-L99
Diseases of the musculoskeletal system and connective tissue	710-739	M00-M99
Diseases of the genitourinary system	580-629	N00-N99
Pregnancy, childbirth and the puerperium	630-676	O00-O99
Certain conditions originating in the perinatal period	760-779	P00-P96
Congenital malformations, deformations and chromosomal abnormalities	740-759	Q00-Q99
Symptoms, signs and abnormal clinical and laboratory findings, not elsewhere classified	780-799	R00-R99
External causes of morbidity and mortality	E800-E999	V01-Y98

*This group includes the diseases of the eye and annexes and the diseases of the ear and the mastoid process.

Table S2. Classification, altitude, area and population by Municipality and monitoring station attributed to each Municipality with its altitude and location.

Municipality					Monitoring station			
**name**	**classification**	**altitude**	**area (km2)**	**population (2011)**	**name**	**altitudine**	**long**	**lat**
Anzola dell'Emilia	Plain/rural	38	36.60	11,851	Padulle Sala Bolognese	25	11.29056300	44.62775200
Argelato	Plain/rural	25	35.10	9,656	Padulle Sala Bolognese	25	11.29056300	44.62775200
Baricella	Plain/rural	11	45.48	6,763	San Pietro Capofiume	11	11.62264000	44.65377600
Bentivoglio	Plain/rural	19	51.11	5,358	Saletto	18	11.44113600	44.63231800
Bologna	Hills/urban	54	140.86	371,337	Bologna Urbana	78	11.32878900	44.50075400
Budrio	Plain/rural	25	120.19	18,008	Mezzolara	20	11.53379300	44.57105300
Calderara di Reno	Plain/rural	30	40.75	13,148	Padulle Sala Bolognese	25	11.29056300	44.62775200
Camugnano	Mountain/rural	692	96.60	2,000	Cottede	794	11.16930800	44.10961700
Casalecchio di Reno	Hills/urban	61	17.33	35,173	Bologna Urbana	286	11.29835500	44.47870100
Castel di Casio	Mountain/rural	533	47.33	3,479	Porretta Terme	352	10.97729800	44.15404100
Castel Maggiore	Plain/urban	29	30.90	17,507	Bologna Urbana	78	11.32878900	44.50075400
Castel d'Aiano	Mountain/rural	805	45.26	1,951	Monteacuto Delle Alpi	900	10.88741300	44.13637700
Castello d'Argile	Plain/rural	23	29.07	6,458	Padulle Sala Bolognese	25	11.29056300	44.62775200
Castenaso	Plain/rural	42	35.73	14,352	Castenaso Villanova	51	11.42939000	44.49023000
Castiglione dei Pepoli	Mountain/rural	691	65.76	5,870	Cottede	794	11.16930800	44.10961700
Crevalcore	Plain/rural	20	102.75	13,527	Sant'Agata Bolognese	18	11.14493100	44.69500200
Gaggio Montano	Mountain/rural	682	58.67	5,066	Monteacuto Delle Alpi	900	10.88741300	44.13637700
Galliera	Plain/rural	14	37.15	5,462	Saletto	18	11.44113600	44.63231800
Granaglione	Mountain/rural	493	39.71	2,232	Porretta Terme	352	10.97729800	44.15404100
Granarolo dell'Emilia	Plain/rural	28	34.37	10,766	Castenaso Villanova	51	11.42939000	44.49023000
Grizzana Morandi	Mountain/rural	547	77.40	3,982	Invaso	460	11.22256600	44.22625300
Lizzano in Belvedere	Mountain/rural	640	85.45	2,309	Monteacuto Delle Alpi	900	10.88741300	44.13637700
Loiano	Hills/rural	714	52.41	4,434	Loiano	741	11.32646200	44.26093300
Malalbergo	Plain/rural	12	53.82	8,771	Saletto	18	11.44113600	44.63231800
Marzabotto	Hills/rural	130	74.53	6,684	Sasso Marconi	275	11.24125100	44.43966800
Minerbio	Plain/rural	16	43.07	8,674	Mezzolara	20	11.53379300	44.57105300
Molinella	Plain/rural	8	127.84	15,651	San Pietro Capofiume	11	11.62264000	44.65377600
Monghidoro	Mountain/rural	841	48.29	3,806	Monghidoro	825	11.32130100	44.21958000
Monte San Pietro	Hills/rural	112	74.69	10,820	Sasso Marconi	275	11.24125100	44.43966800
Monterenzio	Hills/rural	207	105.26	5,853	Bologna San Luca	286	11.29835500	44.47870100
Monzuno	Mountain/rural	621	65.01	6,133	Invaso	460	11.22256600	44.22625300

Ozzano dell'Emilia	Hills/rural	66	64.95	12,870	Castenaso Villanova	51	11.42939000	44.49023000
Pianoro	Hills/rural	200	107.13	16,890	Bologna San Luca	286	11.29835500	44.47870100
Pieve di Cento	Plain/rural	18	15.94	6,895	Padulle Sala Bolognese	25	11.29056300	44.62775200
Porretta Terme	Mountain/rural	349	33.93	4,735	Porretta Terme	352	10.97729800	44.15404100
Sala Bolognese	Plain/rural	25	45.64	8,245	Padulle Sala Bolognese	25	11.29056300	44.62775200
San Benedetto Val di Sambro	Mountain/rural	602	66.47	4,393	Invaso	460	11.22256600	44.22625300
San Giorgio di Piano	Plain/rural	21	30.43	8,201	Saletto	18	11.44113600	44.63231800
San Giovanni in Persiceto	Plain/rural	21	114.41	26.992	Padulle Sala Bolognese	25	11.29056300	44.62775200
San Lazzaro di Savena	Hills/urban	62	44.72	31,091	Bologna Urbana	78	11.32878900	44.50075400
San Pietro in Casale	Plain/rural	17	65.86	11,736	Saletto	18	11.44113600	44.63231800
Sant'Agata Bolognese	Plain/rural	21	34.79	7,140	Sant'Agata Bolognese	18	11.14493100	44.69500200
Sasso Marconi	Hills/rural	128	96.45	14,545	Sasso Marconi	275	11.24125100	44.43966800
Valsamoggia*	Hills/rural	182	178.13	30,025	Sasso Marconi	275	11.24125100	44.43966800
					Ca' Bortolani	691	11.08454800	44.34680400
Vergato	Mountain/rural	193	59.94	7,642	Vergato	193	11.11312800	44.28779600
Zola Predosa	Hills/rural	74	37.75	18,193	Zola Predosa	65	11.20005900	44,49614200

*it was created in 2014 by the union of 5 Municipalities.

Table S3. Psychiatric diagnosis group according to ICD9-CM.

Psychiatric diagnosis in group	ICD9-CM
Schizophrenia and other functional psychosis	295*, 297*, 298* (escl. 298.0), 299* (escl. 299.0, 299.00, 299.01)
Mania and bipolar affective disorders	296.0*, 296.1*,296.4*, 296.5*, 296.6*, 296.7, 296.8* (escl. 296.82),
Depression	296, 296.2*, 296.3*, 296.82, 296.9*, 298.0, 300.4, 309.0, 309.1, 309.1, 311
Neurotic disorders	300* (escl. 300.4), 306*, 307.4*, 307.8*, 307.9, 308*, 309.2* (escl. 309.28), 309.8*, 316
Disorders of personality and behavior	301*, 302*, 312*
Alcoholism and substance abuse	291*, 292*, 303*, 304*, 305*
other	307* (escl 307.4*, 307.8*, 307.9*), 309, 309.3, 309.4, 309.5*, 309.9, 313*, 314*, 315*
